# Complete genome sequence of *Stenotrophomonas maltophilia* complex strain STEN00241

**DOI:** 10.1128/mra.01053-23

**Published:** 2023-12-22

**Authors:** Cristian V. Crisan, Rebecca P. Duncan, Daria Van Tyne, Joanna B. Goldberg

**Affiliations:** 1 Department of Pediatrics, Division of Pulmonary, Asthma, Cystic Fibrosis, and Sleep, Emory University School of Medicine, Atlanta, Georgia, USA; 2 Emory+Children's Center for Cystic Fibrosis and Airway Disease Research, Emory University School of Medicine, Atlanta, Georgia, USA; 3 Division of Infectious Diseases, University of Pittsburgh School of Medicine, Pittsburgh, Pennsylvania, USA; University of Maryland School of Medicine, Baltimore, Maryland, USA

**Keywords:** *Stenotrophomonas*, genome analysis

## Abstract

We report here the complete, closed genome sequence of *Stenotrophomonas maltophilia* complex strain STEN00241, which was isolated from patient sputum. The genome is a single contig with a total length of 4,751,329 nucleotides and a GC content of 66.5%.

## ANNOUNCEMENT

*Stenotrophomonas maltophilia* is a Gram-negative, multidrug-resistant, emerging bacterial pathogen (1–3). The *S*. *maltophilia* complex consists of 23 lineages, and isolates from the same linage share >95% average nucleotide identity (ANI) (4).

*S. maltophilia* complex strain STEN00241 was cultured from patient sputum by the University of Pittsburgh Medical Center Clinical Microbiology Laboratory following standard procedures. The isolate was identified by MALDI-TOF and confirmed through whole genome sequencing (5). The Institutional Review Board approved and exempted the project from informed consent because no direct patient contact was made. A STEN00241 colony was inoculated in liquid LB medium and incubated at 37°C for ~10 hours. Genomic DNA was purified using the Promega Wizard HMW DNA Extraction Kit (following the manufacturer’s instructions) and sequenced at SeqCenter (Pittsburgh, PA, USA) on a MinION (FLO-MIN106) flowcell with R9.4.1 chemistry. Samples were prepared for sequencing using Oxford Nanopore’s “Genomic DNA by Ligation” Kit (SQK-LSK109) and protocol. No shearing or size selection steps were performed during library preparation. Base calling was conducted using Guppy (v5.0.16 in high-accuracy base calling mode; Guppy is available to Oxford Nanopore customers at https://community.nanoporetech.com). The read quality was assessed in NanoPlot (v.1.32.1), and reads were filtered using Filtlong (v.0.2.1) (https://github.com/rrwick/Filtlong) to a minimum length of 1,000 bps. The 10% worst-quality reads were discarded, and the “–target_bases” parameter was set to 500,000,000, resulting in 42,083 reads with an average read quality of 12.3, an average read length of 9,137, and an N50 of 18,919. A consensus genome was assembled using Trycycler (v.0.5.3) (6). Filtered reads were subsampled using “Trycycler subsample” into 12 subsets for a target genome size of 5 Mbps. Four independent read subsets were assembled with either Miniasm (v.0.3-r179) (7) and Minipolish (v.0.1.3) (8), Flye (v.2.9-b1768) (9), or Raven (v.1.8.1) (10). Contigs from these assemblies were clustered and assigned read depths using “Trycycler cluster” by aligning the original filtered reads to each clustered contig. “Trycycler reconcile” was run on the only cluster that contained a single contig from each assembly. Contigs from the reconciled cluster were aligned with MUSCLE using “Trycycler msa.” Original filtered reads were assigned to this cluster using “Trycycler partition” with default minimum aligned length and minimum read coverage parameters (11). Unassigned reads were discarded. Pairwise circularization of the assembly was performed and assessed in Trycycler (v.0.5.3) by rotating each circular contig to *dnaA* (6). Aligned contigs were used to generate a consensus assembly with “Trycycler consensus,” which was polished using Medaka (v.1.6.1) (https://github.com/nanoporetech/medaka) and with Illumina reads (SRR13733231) (5) using Pilon (v.1.24) (12). The genome was annotated with the NCBI Prokaryotic Genome Annotation Pipeline (v.6.6). Default parameters were used for all software unless otherwise specified.

The STEN00241 genome has 4,751,329 nucleotides (66.5% GC content) and 4,340 predicted genes. The ANI between STEN00241 and the *S. maltophilia* 810-2 type strain (GCF_900186865.1) is ~90% (13), and the ANI between STEN00241 and Sm294 (ERR1974504) (from the Sm13 *S. maltophilia* complex lineage) is >99.9% (4). The CARD Resistance Gene Identifier (14) detected putative antibiotic resistance genes in STEN00241 (Table 1).

**TABLE 1:** STEN00241 genes encoding putative antibiotic resistance proteins. The STEN00241 genome was analyzed using the Comprehensive Antibiotic Resistance Database (CARD) Resistance Gene Identifier. “Strict” prediction gene names and genomic positions are displayed below

STEN00241 putative genes encoding antibiotic proteins		
Antibiotic resistance ontology term	Gene start position (nucleotides)	Gene end position (nucleotides)
*adeF*—membrane fusion protein of multidrug efflux complex	1,720,233	1,723,403
*qacJ*—small multidrug resistance antibiotic efflux pump	3,251,237	3,251,569
*smeR*—responder component of a two-component regulatory system modulating antibiotic efflux	4,510,293	4,510,982

## Data Availability

The genome sequence of *S. maltophilia* complex STEN00241 has been deposited at the National Center for Biotechnology Information (NCBI) with the assembly accession number CP136247. The BioProject accession number is PRJNA1023947, and the BioSample accession number is SAMN37685180. The raw Oxford Nanopore reads are available under the accession number SRR26361329.
